# Safety and efficacy of early oral switch in Enterobacterales bacteremia: a systematic review and meta-analysis

**DOI:** 10.12701/jyms.2026.43.12

**Published:** 2026-01-07

**Authors:** Rafael Levandowski, Tae Yoon Hwang, Sangwoon Bae, Kyeong-Soo Lee

**Affiliations:** 1 Department of Preventive Medicine and Public Health, Yeungnam University College of Medicine, Daegu, Korea; 2 Department of Internal Medicine, Yeungnam University College of Medicine, Daegu, Korea

**Keywords:** Bacteremia, Early oral switch, *Enterobacteriaceae*, Meta-Analysis, Step-down therapy

## Abstract

**Background:**

Early oral switch (EOS) has been proposed as an alternative to prolonged intravenous (IV) therapy for Enterobacterales bacteremia; however, its safety and effectiveness have not been clearly established. This systematic review and meta-analysis evaluated whether EOS reduces treatment failure in uncomplicated Enterobacterales bacteremia and examined how the timing of switching affects outcomes.

**Methods:**

We conducted a systematic review and random-effects meta-analysis of randomized controlled trials and observational studies that compared EOS with continued IV therapy in adults with uncomplicated Enterobacterales bacteremia. Pooled risk ratios (RRs) with 95% confidence intervals (CIs) were calculated using random-effects models. The primary outcome was treatment failure by ≤90 days. Prespecified analyses evaluated the timing of the switch.

**Results:**

Across 10 studies that met the inclusion criteria, EOS was associated with lower treatment failure than continued IV therapy (RR, 0.72; 95% CI, 0.58–0.89; I²=26%). Switching within 4 days reduced the failure (RR, 0.58; 95% CI, 0.44–0.76; I²=0%), whereas switching after 4 days showed no clear advantage (RR, 0.87; 95% CI, 0.71–1.06; I²=0%). No evidence of small study effects was observed.

**Conclusion:**

EOS was associated with a lower risk of treatment failure than prolonged IV therapy, with the greatest benefit observed when the switch occurred within 4 days. These findings should be interpreted with clinical caution given that most of the evidence is observational.

## Introduction

Enterobacterales are among the leading causes of bloodstream infections in adults, responsible for more than half of gram-negative bacteremia cases, and rank among the most frequent etiologies of sepsis [1]. The most common sources are urinary tract, hepatobiliary system, and intra-abdominal infections [1,2]. These infections are also increasingly complicated by antimicrobial resistance, with extended-spectrum β-lactamase and carbapenem-resistant strains reported at rising frequencies [2]. Case fatality rates remain high and frequently exceed 10%, particularly in patients with significant comorbidities.

Intravenous (IV) antibiotics remain the standard treatment, and guidelines recommend 7 to 14 days of therapy initiated with an IV phase [3]. However, prolonged IV therapy extends hospitalization, increases costs, and exposes patients to catheter-related complications, such as phlebitis, occlusion, and secondary bloodstream infections [4,5]. These limitations have prompted interest in earlier transition to oral therapy once patients are stable and the infection source is controlled. Observational studies have suggested that this early oral switch (EOS) is safe, particularly in urinary tract or other adequately controlled infections; however, its implementation is inconsistent [6-8]. Reported switch rates range from 20% to >50%, and definitions of “early” vary from day 3 to day 7. Moreover, many clinicians delay transition, assuming that IV therapy is more reliable, even in patients who are already stable.

However, supporting evidence remains limited. Most studies are observational, randomized controlled trials (RCTs) are rare, and no synthesis has provided firm conclusions on the safety of EOS or the influence of switch timing [9-11]. To address these gaps, we conducted a systematic review and meta-analysis comparing EOS and continued IV therapy in adults with uncomplicated Enterobacterales bacteremia as the primary objective.

## Methods

**Ethics statement:** Not applicable.

### 1. Data sources and search strategy

The conduct and reporting of this review adhered to the Cochrane Handbook for Systematic Reviews of Interventions. We searched PubMed, Embase, and Scopus for publications from database inception until September 2025, and screened the reference lists of eligible articles and related reviews. Our search algorithm combined controlled vocabulary and free-text terms using structured Boolean operators: (“Enterobacterales” OR “Enterobacteriaceae” OR “Gram-negative bacteremia” OR “bloodstream infection”) AND (“oral step-down” OR “oral switch” OR “early oral transition” OR “sequential therapy”) AND (“fluoroquinolones” OR “trimethoprim-sulfamethoxazole” OR “TMP-SMX” OR “oral β-lactams” OR “cephalosporins” OR “high-bioavailability oral agents”). No language filter was used in the search. Although the search strategy did not rely on a fully optimized Medical Subject Headings-based structure, the selected terms were intended to maximize the retrieval of relevant studies.

### 2. Study selection

The eligibility criteria were defined according to a prespecified PICOS (population, intervention, comparator, outcome) framework for EOS versus IV. We included studies that enrolled adults with uncomplicated Enterobacterales bacteremia who achieved clinical stability after initial management and had adequate source control. EOS was defined as transition to oral therapy within 7 days of initiating effective IV treatment. The eligible study designs were RCTs and observational cohorts comparing EOS and IV, which constituted the primary research aim.

The primary outcome was treatment failure within 90 days, and secondary outcomes were extracted when available. Only human studies published in English were included. “Uncomplicated” bacteremia was defined *a priori* as an infection not involving endocarditis, the osteoarticular system, the central nervous system, uncontrolled foci, persistent bacteremia beyond 72 hours, severe immunosuppression, or indwelling prosthetic material requiring prolonged therapy. As this classification cannot be reliably applied at the search level, it was implemented during full-text screening.

Finally, studies were eligible only if outcomes specific to Enterobacterales bacteremia were extractable. In studies that included mixed gram-negative organisms, we extracted Enterobacterales-specific data when subgroup results were provided. When stratification was not available, studies were included only if Enterobacterales represented the majority of isolates, and no organism-specific differences in management were expected. This approach ensured that the pooled estimates reflected the outcomes predominantly attributable to Enterobacterales.

### 3. Data extraction and quality assessment

All references were managed using the EndNote 20 software (Clarivate, Philadelphia, PA, USA). Screening and data extraction were conducted independently by two reviewers using a standardized form. Titles, abstracts, and full texts were reviewed in duplicate, and discrepancies at any stage were resolved by discussion and consensus, with the involvement of a third reviewer when necessary. For the analysis, we collected data on the number of treatment failures and the total number of patients in each group. Treatment failure was defined *a priori* as any of the following within the longest reported follow-up period (≤90 days): all-cause mortality, microbiologically or clinically documented recurrence of the same pathogen, infection-related readmission, or the need to restart or escalate IV antibiotics. When mortality was the only reported outcome, survivors within the follow-up window were considered treatment successes. The risk of bias was assessed independently by two reviewers using the ROBINS-I tool for non-randomized studies and the RoB 2 tool for RCTs, with discrepancies resolved by consensus. Visualizations were generated using the *robvis* package [12].

### 4. Statistical analysis

All analyses were performed using R software version 4.5.1 (R Foundation for Statistical Computing, Vienna, Austria) using the *meta, metafor*, and *bayesmeta* packages. The primary meta-analysis was conducted by comparing EOS and IV therapies. For each study, the risk ratios (RRs) for treatment failure within 90 days were calculated from raw event counts or extracted directly when reported. Given the clinical and methodological heterogeneity across studies, including differences in design, patient characteristics, and definitions of EOS, we assumed that the true effects could vary and therefore applied random-effects models. Random-effects Mantel–Haenszel models were used and between-study variance (τ²) was estimated using the restricted maximum likelihood method with Hartung–Knapp adjustment. Effect estimates are presented with 95% confidence intervals (CIs), heterogeneity statistics (I² and τ²), and prediction intervals when appropriate.

The timing of oral transition was treated as a prespecified subgroup moderator. When studies reported a switching window, the midpoint of that interval was used to classify cohorts into ≤4 days or >4 days. Separate random-effects models were fitted for each subgroup, and a test for subgroup differences was performed to assess whether timing modified the pooled effect. We also performed prespecified subgroup analyses stratified by outcome definition (mortality-only, recurrence-only, and composite endpoints) to avoid pooling outcomes with different clinical interpretations.

A Bayesian random-effects meta-analysis was performed as a sensitivity assessment, using a normal prior (mean, 0; standard deviation, 1.0) for the overall log RR and a half-normal prior (scale, 0.5) for τ, implemented with the *bayesmeta* package. Posterior distributions were summarized using posterior means and 95% credible intervals (CrIs). The agreement between the Bayesian and frequentist estimates was used to evaluate robustness of the model specification.

Potential publication bias was assessed using contour-enhanced funnel plots, Egger’s regression as the primary test for small study effects, and Begg’s rank correlation as a complementary approach.

## Results

### 1. Literature search and selection of studies

**Fig. 1.**
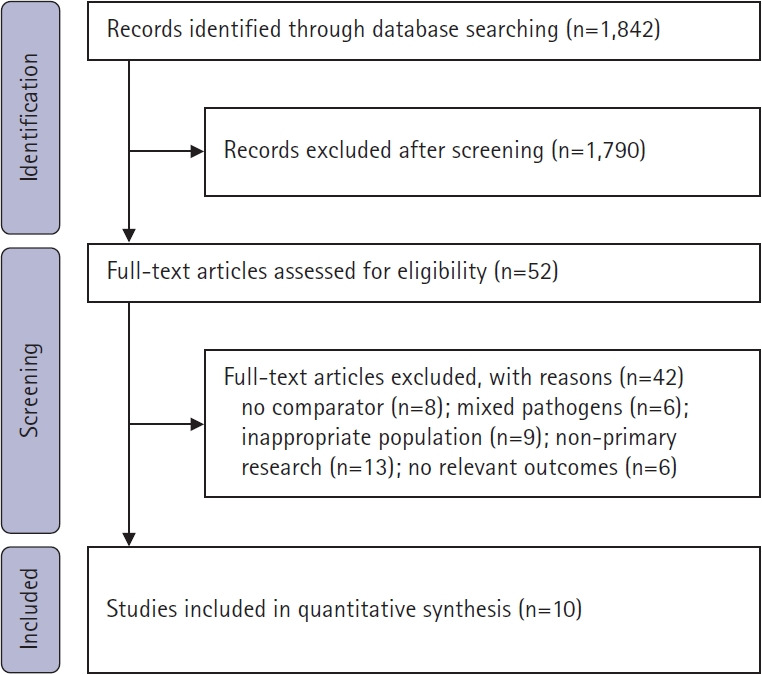
Flowchart of study selection for the meta-analysis.

The search yielded 1,842 records. After removing duplicates and screening titles and abstracts, 52 full texts were selected for review. A total of 42 were excluded due to the absence of a full IV comparator (n=8), mixed pathogen populations without separable Enterobacterales data (n=6), inappropriate infection focus (n=9), non-primary research (n=13), or a lack of extractable outcomes (n=6). Ten studies met the eligibility criteria (Fig. 1).

### 2. Study characteristics

The included studies encompassed 5,565 adults with uncomplicated Enterobacterales bacteremia. The evidence base consisted of two RCTs and eight cohort studies conducted in single and multicenter settings (Table 1 [5,6,9-11,13-17]). All 10 studies contributed data to the EOS versus IV analysis. Most infections originated from urinary sources, but the definitions of EOS and follow-up durations varied substantially across studies, reflecting the heterogeneity observed in routine clinical practice.

### 3. Publication bias

Publication bias was evaluated using funnel plots (Supplementary Figs. 1,2) and statistical analyses. Egger’s regression did not identify evidence of small study effects (*p*=0.27 using *meta* and *p*=0.50 using *metafor*), and Begg’s test was also not significant (*p*=0.93). Trim-and-fill identified one potentially missing study, with an adjusted estimate (RR, 0.73; 95% CI, 0.58–0.91) that remained close to the original result. The available tests did not indicate a meaningful publication bias, although the inference was limited by the small number of eligible studies.

### 4. Risk of bias assessment

**Fig. 2.**
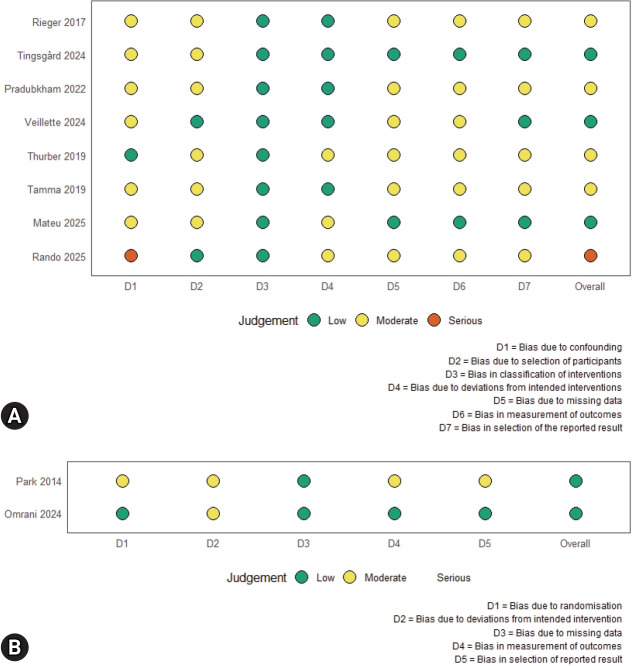
Risk of bias assessment across included studies. (A) Risk of bias summary for non-randomized studies (ROBINS-I). (B) Risk bias summary for randomized studies (RoB 2).

The risk of bias evaluations is summarized in Fig. 2. Two RCTs [9,13] were assessed with RoB 2 and showed low risk or some concerns. Most cohorts were rated as moderate risk using ROBINS-I, primarily because of residual confounding and deviations from the intended interventions. One study [11] was judged to have a serious risk owing to insufficient adjustment and incomplete handling of confounding. No study was excluded based on the risk of bias ratings.

### 5. Early oral switch versus continued intravenous therapy

**Fig. 3.**
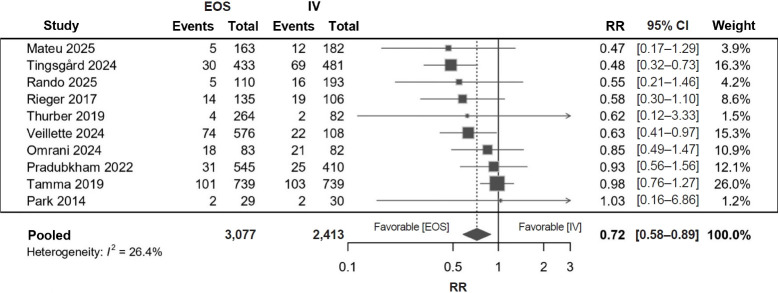
Meta-analysis of early oral switch versus intravenous therapy for treatment failure. Risk ratios (RRs) <1 indicate fewer failures with EOS. EOS, early oral switch; IV, intravenous; CI, confidence interval.

**Fig. 4.**
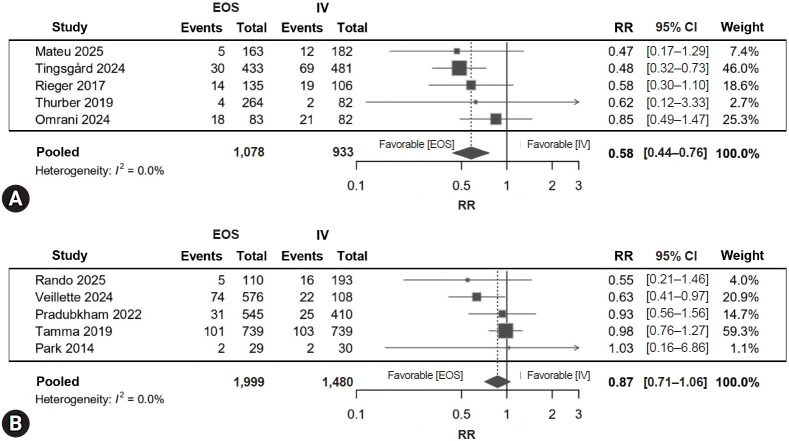
Timing of early oral switch and treatment failure. (A) Switch ≤4 days. (B) Switch >4 days. Risk ratios (RRs) <1 indicate fewer failures with EOS. EOS, early oral switch; IV, intravenous; CI, confidence interval.

Ten studies including 5,565 patients contributed to the comparison of EOS and continued IV therapy (Fig. 3). EOS was associated with a lower risk of treatment failure (RR, 0.72; 95% CI, 0.58–0.89; I²=26%; *p*=0.0056), corresponding to a 29% relative risk reduction (approximately 3–4 fewer failures per 100 patients). Regarding the timing of transition (Fig. 4), patients who switched to EOS within 4 days had fewer failures (RR, 0.58; 95% CI, 0.44–0.76; I²=0%; *p*=0.010; 4–5 fewer failures per 100 patients), whereas switching after 4 days showed no significant difference compared with continued IV therapy (RR, 0.87; 95% CI, 0.71–1.06; I²=0%; *p*=0.170). The between-subgroup difference was statistically significant (*p*=0.014) (Table 2). Leave-one-out influence analyses showed that no individual study materially altered the pooled estimate and alternative continuity corrections yielded similar results.

### 6. Bayesian analyses

The posterior mean RR was 0.72 (95% CrI, 0.55–0.93). The close agreement between the Bayesian and frequentist estimates indicates that the overall conclusions were not sensitive to the modeling assumptions.

### 7. Additional sensitivity considerations

Additional sensitivity analyses supported the stability of the primary findings. When studies were stratified by outcome definition (mortality-only, recurrence-only, and composite endpoints), the direction of the association consistently favored EOS, although the effect sizes varied across strata, ranging from larger relative effects in mortality-only outcomes to more attenuated, nonsignificant estimates in composite endpoints. No meaningful shifts were observed when studies with a higher risk of bias were excluded, and no single study disproportionately influenced the pooled estimate. Detailed subgroup results are presented in Supplementary Table 1.

## Discussion

This meta-analysis synthesized evidence from 10 studies and 5,565 adult patients, providing a comprehensive assessment of EOS for Enterobacterales bacteremia. This fills the gap left by previous cohorts [8,14] that reported no excess mortality with EOS; however, their conclusions were limited to non-inferiority and constrained by sample size, follow-up, or analytical methods. By pooling data across studies, we found a consistent 29% relative reduction in treatment failure, a benefit not evident in individual reports. Moreover, our findings contrast with those of Veillette et al. [15], who concluded equivalence between EOS and prolonged IV therapy. When integrated across studies, evidence favors EOS in patients who are clinically stable with adequate source control measures, highlighting the value of meta-analysis in detecting effects that are not evident in single cohorts.

The timing of transition emerged as another relevant factor. Switching within 4 days was associated with significantly fewer failures, whereas later switching did not show the same advantage. Notably, the outcomes after day 4 were not worse than those of continued IV therapy, indicating that transition remains a safe option even beyond the early window. These results align with observations from previous reports [5,6,16] and complement the Danish registry analysis [10], which highlighted that early switchers were often more stable at baseline. The data indicate that the advantage is most pronounced when switching within 4 days, although clinical stability, rather than calendar time, appears to be a more relevant criterion for transition.

However, it is important to note that the definitions of treatment failure varied across studies, ranging from mortality alone to composites, including relapse, readmission, or therapy modification. This heterogeneity complicates comparisons, but harmonizing endpoints in our analysis still produced robust results, and sensitivity analyses confirmed the consistency of the benefits of EOS.

Several studies have also compared different oral treatment regimens after EOS. Across this literature, fluoroquinolone or trimethoprim-sulfamethoxazole regimens are more frequently associated with lower failure or recurrence rates than oral β-lactam regimens [8,11,18]. Concerns regarding higher recurrence with oral β-lactams have been raised in multiple observational cohorts [15,19]. At the same time, other studies have suggested that optimized β-lactam dosing attenuates these differences and improves outcomes [7,20]. These findings indicate that oral regimen selection may influence the outcomes after EOS, although the available evidence remains heterogeneous and largely observational.

Although our analysis focused on Enterobacterales, complementary data from *Staphylococcus aureus* bacteremia point in the same direction. The ongoing *Staphylococcus aureus* Network Adaptive Platform trial is testing oral switch guided by stability and source control, while European stewardship initiatives show that structured protocols can standardize these decisions and reduce unnecessary IV exposure. Even selected low-risk *S. aureus* cohorts treated with oral β-lactams have reported low relapse, reinforcing that outcomes depend more on patient selection than on the regimen itself, although in many studies, high-bioavailability agents have shown to be more reliable options for Enterobacterales [21-23].

Moreover, although not directly assessed in this study, EOS reduces the need for prolonged venous access, lowers the risk of catheter-related complications, and enables earlier discharge [5,9]. These benefits translate into lower healthcare costs and improved patient comfort, underscoring that EOS is a safe and pragmatic strategy.

Before translating these findings into clinical practice, several limitations should be acknowledged. Most of the included studies were observational and, therefore, subject to residual confounding and immortal time bias. Only two RCTs were available, which were both too small and underpowered to detect modest differences. Outcome definitions and follow-up periods varied, and data on resistant pathogens or non-urinary tract sources were scarce, limiting the external validity.

The findings of this review indicate that EOS is a safe and effective strategy for adults who are clinically stable with uncomplicated Enterobacterales bacteremia when adequate source control is achieved. The consistency of effects across heterogeneous designs, including cohorts and the two available RCTs, supports its applicability in routine practice while also underscoring gaps where evidence remains limited. Future RCTs should refine the optimal timing of transition and evaluate outcomes in non-urinary tract and resistant infections to better guide stewardship efforts.

## Figures and Tables

**Table 1.** Characteristics of included studies

Study (year)	Country	Number	Design
Rieger et al. [6] (2017)	USA	241	Cohort
Park et al. [13] (2014)	Korea	59	RCT
Tingsgård et al. [10] (2024)	Denmark	914	Cohort
Omrani et al. [9] (2024)	Multicenter^a)^	165	RCT
Pradubkham et al. [17] (2022)	Thailand	955	Cohort
Veillette et al. [15] (2024)	USA	759	Cohort
Thurber et al. [5] (2019)	USA	346	Cohort
Tamma et al. [14] (2019)	USA	1,478	Cohort
Mateu et al. [16] (2025)	Spain	345	Cohort
Rando et al. [11] (2025)	Multicenter^a)^	303	Cohort

RCT, randomized controlled trial.

a) Conducted at two or more recruitment centers.

**Table 2.** Absolute risk estimates from pooled analyses of early oral step-down versus intravenous therapy (treatment failure)

Aspect	Window	RR (95% CI)	I^2^(%)	*p-*value	Failure (per 100 patients)
Treatment failure	≤7 days (n=10)	0.72 (0.58–0.89)	26	0.006	3–4 fewer failures
Timing of switch	≤4 days (n=5)	0.58 (0.44–0.76)	0	0.01	4–5 fewer failures
	>4 days (n=5)	0.87 (0.71–1.06)	0	0.17	No clear difference

RR, risk ratio; CI, confidence interval. The *p*-values were calculated at the meta-analytic level and represent tests of the pooled random-effects estimates. Failure calculated per 100 patients.

## Supplementary materials

Supplementary Tables 1 and Supplementary Figs. 1–3 can be found at https://doi.org/10.12701/jyms.2026.43.11.

Supplementary Fig. 1. Funnel plot. Each dot represents one study. The x-axis shows the log risk ratio (log RR) and the y-axis the standard error (larger studies appear higher). The vertical dotted line marks no effect (log RR=0). The white triangular region depicts the expected 95% region under no small-study effects. Visual inspection shows no marked asymmetry, suggesting no strong publication/small-study bias for either analysis. EOS, early oral switch; IV, intravenous.

Supplementary Fig. 2. Funnel plot (trim-and-fill) assessing publication bias. Early oral switch vs. intravenous-only therapy. Visual inspection showed no clear asymmetry. Trim-and-fill imputed at most one study, but pooled estimates remained stable. SE, standard error; RR, risk ratio.

Supplementary Table 1. Outcome-stratified subgroup analyses by definition of treatment failure
