# Supplementary material for: Safety and efficacy of early oral switch in Enterobacterales bacteremia: a systematic review and meta-analysis
Source: J Yeungnam Med Sci. 2026 Jan 7;43:12. doi: 10.12701/jyms.2026.43.12 (PMC12887126; doi:10.12701/jyms.2026.43.12)
Supplement: Supplementary Fig. 1. — Funnel plot. Each dot represents one study. The x-axis shows the log risk ratio (log RR) and the y-axis the standard error (larger studies appear higher). The vertical dotted line marks no effect (log RR=0). The white triangular region depicts the expected 95% region under no small-study effects. Visual inspection shows no marked asymmetry, suggesting no strong publication/small-study bias for either analysis. EOS, early oral switch; IV, intravenous. [file jyms-2026-43-12-Supplementary-Fig-1.pdf]

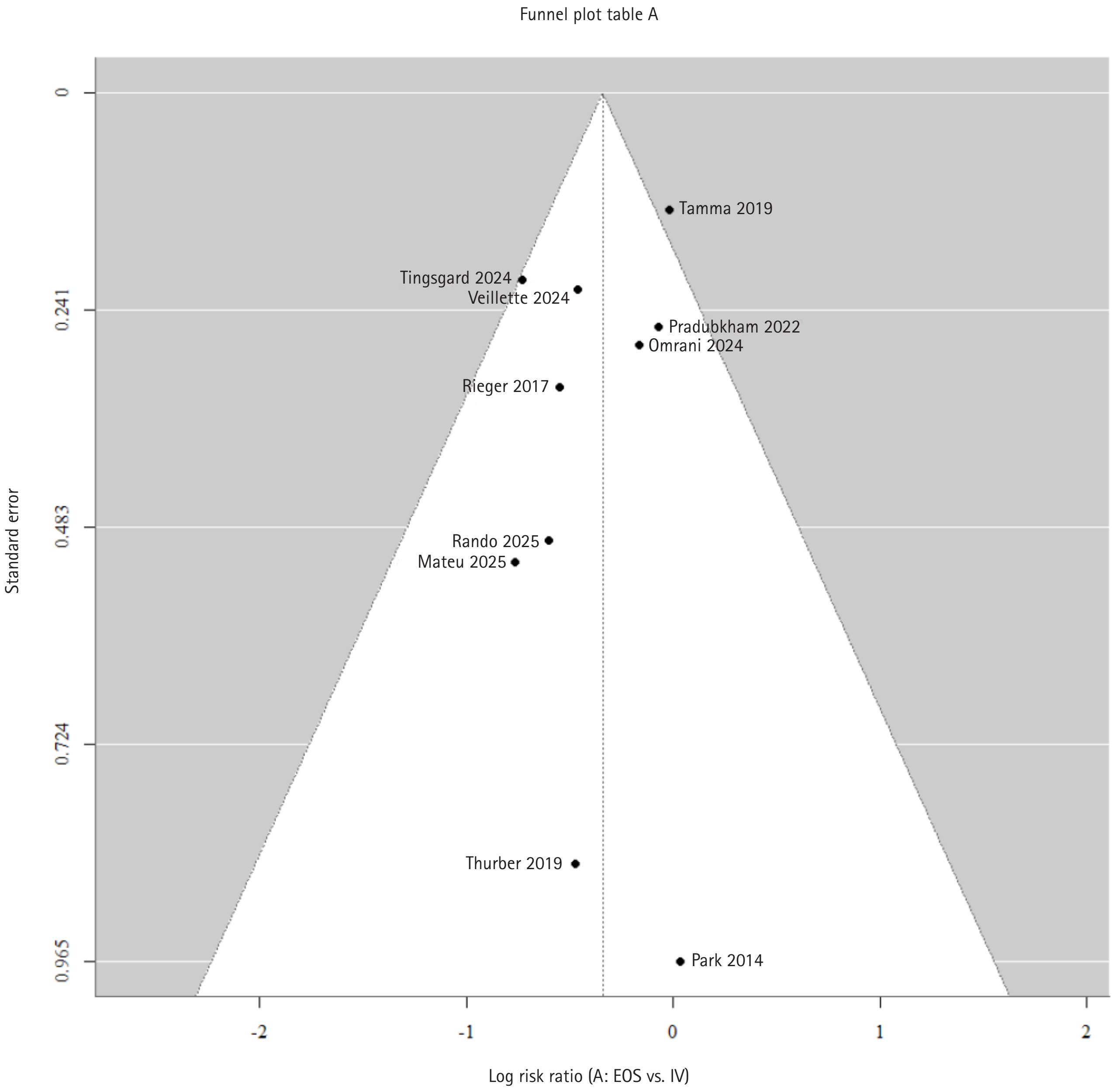

Supplementary Fig. 1. Funnel plot. Each dot represents one study. The x-axis shows the log risk ratio (log RR) and the y-axis the standard error (larger studies appear higher). The vertical dotted line marks no effect (log RR = 0). The white triangular region depicts the expected 95% region under no small-study effects. Visual inspection shows no marked asymmetry, suggesting no strong publication/small-study bias for either analysis.
EOS, early oral switch; IV, intravenous.
