# Supplementary material for: Safety and efficacy of early oral switch in Enterobacterales bacteremia: a systematic review and meta-analysis
Source: J Yeungnam Med Sci. 2026 Jan 7;43:12. doi: 10.12701/jyms.2026.43.12 (PMC12887126; doi:10.12701/jyms.2026.43.12)
Supplement: Supplementary Fig. 2. — Funnel plot (trim-and-fill) assessing publication bias. Early oral switch vs. intravenous-only therapy. Visual inspection showed no clear asymmetry. Trim-and-fill imputed at most one study, but pooled estimates remained stable. SE, standard error; RR, risk ratio. [file jyms-2026-43-12-Supplementary-Fig-2.pdf]

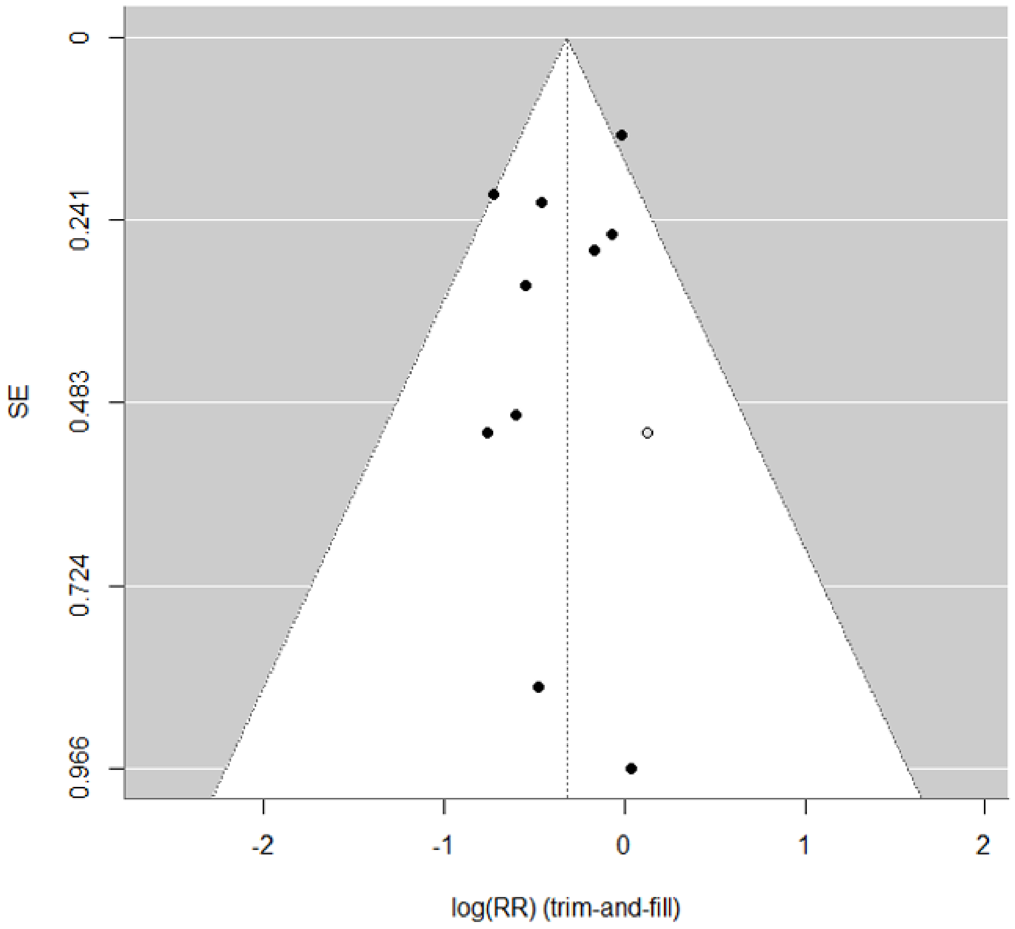

Supplementary Fig. 2. Funnel plot (trim-and-fill) assessing publication bias. Early oral switch vs. intravenous-only therapy. Visual inspection showed no clear asymmetry. Trim-and-fill imputed at most one study, but pooled estimates remained stable. SE, standard error; RR, risk ratio.
