# Supplementary material for: Safety and efficacy of early oral switch in Enterobacterales bacteremia: a systematic review and meta-analysis
Source: J Yeungnam Med Sci. 2026 Jan 7;43:12. doi: 10.12701/jyms.2026.43.12 (PMC12887126; doi:10.12701/jyms.2026.43.12)
Supplement: Supplementary Table 1. — Outcome-stratified subgroup analyses by definition of treatment failure [file jyms-2026-43-12-Supplementary-Table-1.pdf]

Supplementary Table 1. Outcome-stratified subgroup analyses by definition of treatment failure

Outcome definition	Studies (k)[a]	Patients (EOS/IV)	Pooled RR (95% CI)	I^2 (%)	*p*-value
Mortality only	1	433/481	0.48 (0.32–0.73)	NA	NA
Recurrence only	2	840/190	0.63 (0.42–0.96)	0	0.03
Composite without mortality	2	680/516	0.77 (0.48–1.21)	23	0.256
Composite including mortality	5	1,124/1,225	0.90 (0.72–1.12)	0	0.346

EOS, early oral switch; IV, intravenous; RR, risk ratio; CI, confidence interval; NA, not applicable.
[a]Indicates the number of studies included in each subgroup analysis.
